# Biological Nanoparticles in Vaccine Development

**DOI:** 10.3389/fbioe.2022.867119

**Published:** 2022-03-23

**Authors:** Stephanie M. Curley, David Putnam

**Affiliations:** ^1^ Meinig School of Biomedical Engineering, Cornell University, Ithaca, NY, United States; ^2^ Smith School of Chemical and Biomolecular Engineering, Cornell University, Ithaca, NY, United States

**Keywords:** vaccines, nanoparticles, VLP, OMV, protein cages

## Abstract

Vaccines represent one of the most successful public health initiatives worldwide. However, despite the vast number of highly effective vaccines, some infectious diseases still do not have vaccines available. New technologies are needed to fully realize the potential of vaccine development for both emerging infectious diseases and diseases for which there are currently no vaccines available. As can be seen by the success of the COVID-19 mRNA vaccines, nanoscale platforms are promising delivery vectors for effective and safe vaccines. Synthetic nanoscale platforms, including liposomes and inorganic nanoparticles and microparticles, have many advantages in the vaccine market, but often require multiple doses and addition of artificial adjuvants, such as aluminum hydroxide. Biologically derived nanoparticles, on the other hand, contain native pathogen-associated molecular patterns (PAMPs), which can reduce the need for artificial adjuvants. Biological nanoparticles can be engineered to have many additional useful properties, including biodegradability, biocompatibility, and are often able to self-assemble, thereby allowing simple scale-up from benchtop to large-scale manufacturing. This review summarizes the state of the art in biologically derived nanoparticles and their capabilities as novel vaccine platforms.

## Introduction

**FIGURE 1:**
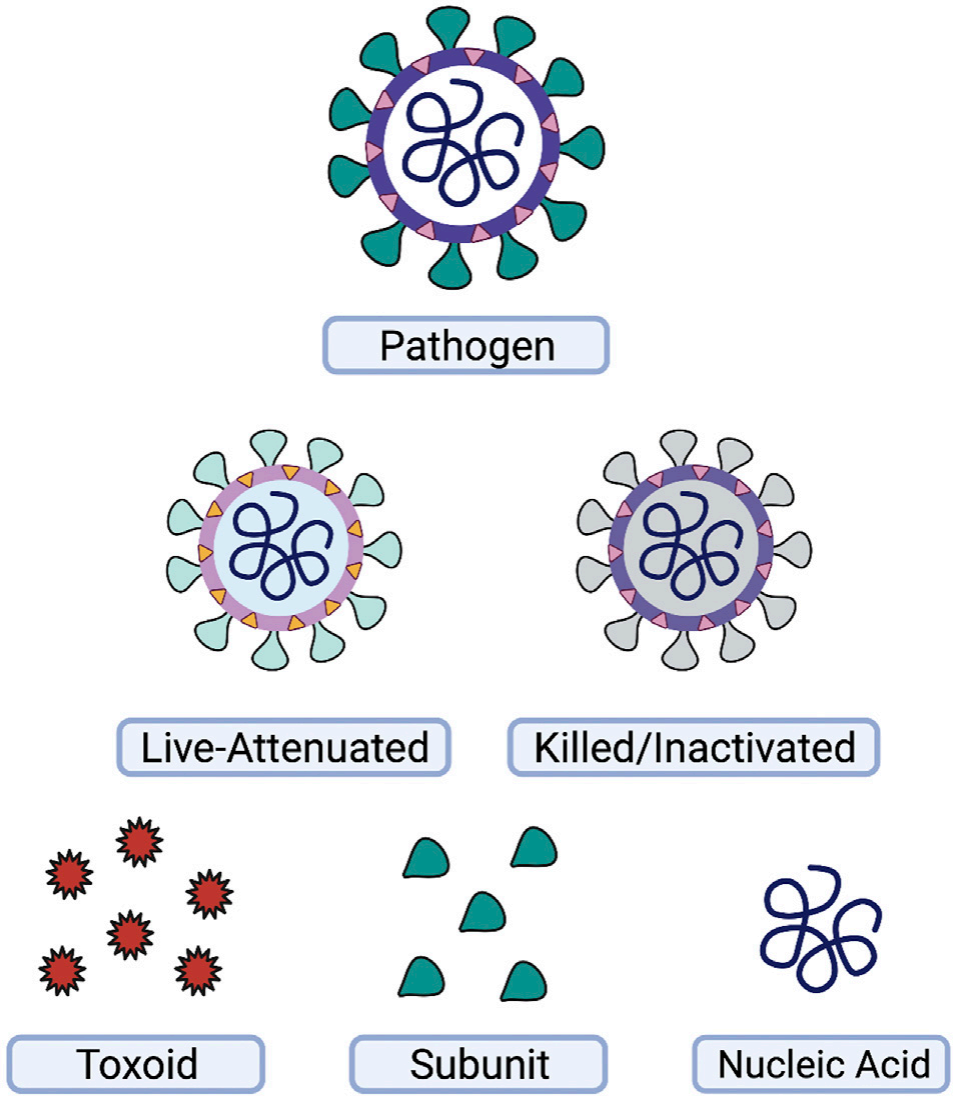
Types of vaccines. A wide variety of vaccines have been used for prevention of disease. Vaccines may be based on the whole-pathogen, such as live-attenuated or killed-inactivated vaccines, or on subsets of the pathogen, such as toxins or protein subunits produced by the pathogen. Nucleic acid vaccines utilizing DNA or RNA are another strategy for vaccine development. This figure was generated using Biorender.com.

Vaccines, arguably, represent one of the most successful preventative health initiatives worldwide. As is evident from the COVID-19 pandemic, vaccines are valuable to not only prevent severe symptoms, hospitalizations, and deaths, but also to prevent subsequent gain of function mutations of the pathogen during replication in the host’s cells. Historically, vaccines are divided into three main categories: live-attenuated, inactivated, and subunit/toxoid (Figure 1).

Live-attenuated vaccines provide good immune memory and neutralizing antibody responses. One reason for their efficiency is that live pathogens display pathogen-associated molecular patterns (PAMPs) on their surface and interior, which are recognized by pattern-recognition receptors, such as toll-like receptors (TLRs) and NOD-like receptors (NLRs) of the innate immune system. Due to their live nature and assorted PAMPs, live vaccines often do not require additional adjuvants or periodic booster shots to re-activate the memory response. Unfortunately, live vaccines can be dangerous to those with weakened immune systems, and as such, these individuals require other vaccine types.

Inactivated vaccines, which are pathogens that have been “killed” with heat or chemical treatment, provide some memory response, but overall require the use of adjuvants, such as aluminum hydroxide, to act as artificial PAMPs, and fully engage the adaptive immune system. While often effective in inducing a robust memory response to an inactivated pathogen, this strategy alone is not always adequate for generating life-long protection. Therefore, a number of inactivated vaccines eventually require use of a booster dose to re-activate the immune system and once again generate a protective response.

Subunit and toxoid vaccines function similarly to inactivated vaccines but are made from a specific component of the pathogen, such as a protein or toxin. These vaccines have a favorable safety profile over the whole-pathogen vaccines mentioned above, as they are unable to replicate, and are non-infectious. On the other hand, as these pathogen-derived antigens represent only one component of the pathogen in many formulations, they are often poorly or non-immunogenic, making adjuvants and booster shots a necessity.

Over the past decade, there has been interest in creating novel vaccines with improved safety and efficacy profiles. These efforts have led to the development of a number of new vaccine platforms, including RNA vaccines which, thanks to record-breaking development on the COVID-19 vaccines, can now be added to the list of licensed human vaccines. The COVID-19 vaccines recently developed by Pfizer-BioNTech and Moderna use synthetic lipid components to encapsulate and protect messenger RNA (mRNA). The mRNA then enters the cells near the injection site and induces them to make a viral protein, which then activates the immune system. Interestingly, the immune response generated from the mRNA vaccines persists for more than 6 months (Doria-Rose et al., 2021; Pegu et al., 2021), but an overall decrease from peak response after 6 months is evident (Naaber et al., 2021; Pegu et al., 2021). In addition to this waning immune response, viral variants have further evaded these moderately efficacious vaccines, making booster doses required (Choi et al., 2021).

**FIGURE 2:**
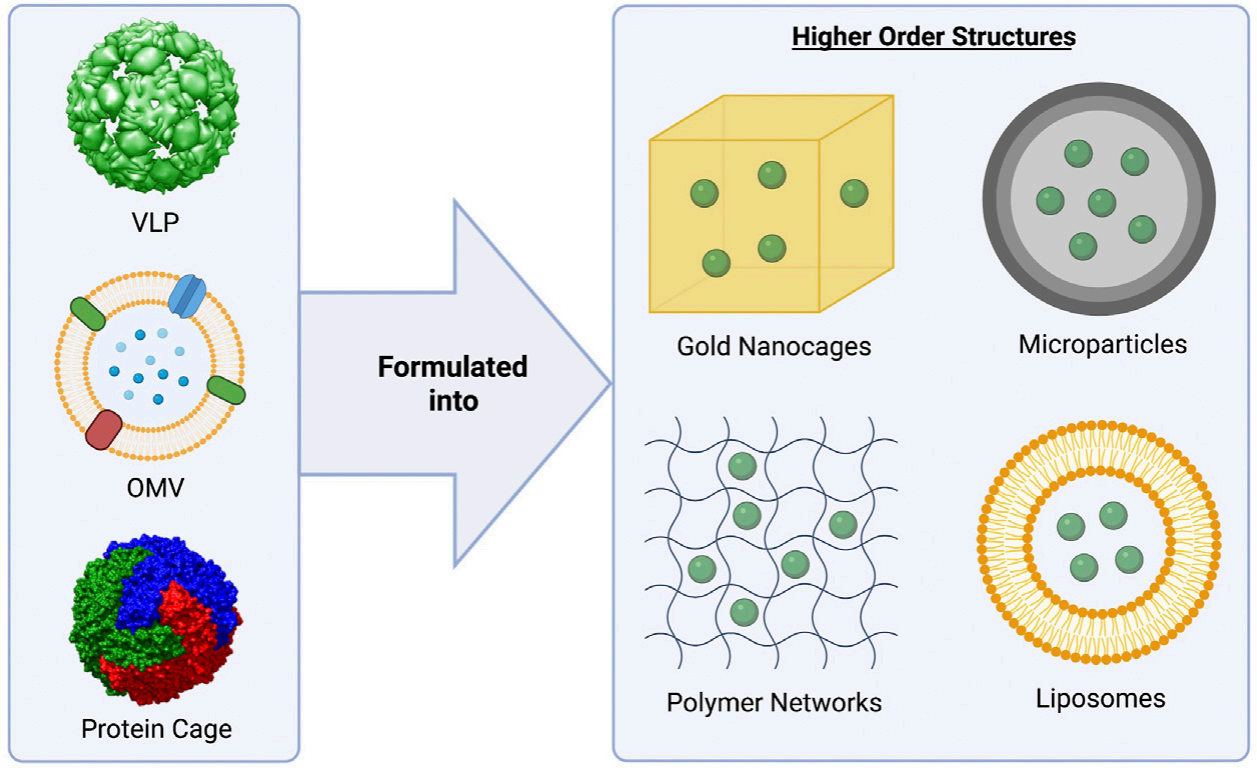
Biologically-derived vaccine platforms highlighted in this review. Virus-like particles (VLPs), outer membrane vesicles (OMVs), and protein cages (ferritin) have been studied extensively as vaccine platforms (left). These biological nanoparticles may be formulated into higher order structures (right) to increase their stability and adjuvanticity, as well as enabling controlled release for use as single-dose vaccine platforms. MS2 bacteriophage was used as a model for the VLP (pdb id. 2MS2 (Golmohammadi et al., 1993)). For the modeling of the ferritin nanocage, the crystal structure of L-ferritin (pdb id. 2fg8 (Wang et al., 2006)) was used. The VLP and nanocage were constructed using UCSF Chimera, developed by the Resource for Biocomputing, Visualization, and Informatics at the University of California, San Francisco, with support from NIH P41-GM103311 (Pettersen et al., 2004). The OMV figure was created using BioRender.com.

Developing universal vaccines capable of generating a long-lasting, protective immune response without utilizing live pathogens has been a challenge. Biologically-derived nanoparticles, such as virus-like particles, outer membrane vesicles, and protein nanocages, may meet this need, as they mimic the structure and function of live pathogens, but are unable to replicate and are not infectious (Figure 2). These nanoparticles naturally contain many of the PAMPs required for full activation of the immune system and often don’t require additional adjuvants. Additionally, as there is no danger that these systems will undergo pathogen reversion, biological nanoparticles may have an improved safety profile over whole-pathogen based vaccines. Due to their native properties, capacity for genetic engineering, and high versatility, these nanoparticle systems have been successfully translated from the benchtop to the clinic, with multiple approved vaccine platforms available to the public, and others currently in clinical trials (Tables 1, 2). In fact, virus-like particles (VLPs) and outer membrane vesicles (OMVs), specifically, have had great success, with four FDA-approved VLP vaccines, and two FDA-approved OMV vaccines available to the public (Table 1). Protein nanocages based on ferritin, three VLP-based vaccines, and one OMV vaccine are currently being investigated for safety and efficacy in clinical trials (Table 2).

**TABLE 1:** FDA-approved biologically-derived nanoparticle vaccines.

Vaccine name	Target pathogen	Type	Company	References
Cervarix^®^	HPV	VLP	GlaxoSmithKline	Harper et al. (2004)
Gardasil^®^	HPV	VLP	Merck & Co.	Villa et al. (2005)
Engerix^®^	HBV	VLP	GlaxoSmithKline	Keating et al. (2003)
Recombivax^®^	HBV	VLP	Merck & Co.	Van Damme et al. (2009)
Bexero^®^	MenB	OMV	Novartis	Giuliani et al. (2006)
PedVaxHIB^®^	Hib	OMV	Merck & Co.	Vella et al. (1990)

**TABLE 2:** Biologically-derived nanoparticle vaccines recruiting or in active clinical trials in the United States.

Type	Target disease	Company/Sponsor	Phase	NCT number
VLP (plant)	Influenza	Medicago	1, 2	NCT04622592
VLP (plant)	SARS-CoV-2	Medicago	2,3	NCT04636697
VLP (recombinant)	Encephalitis	SRI International	1	NCT03776994
VLP (recombinant)	Chikungunya	Emergent BioSolutions	2, 3	NCT05065983, NCT05072080
OMV	Gonorrhea	NIAID	2	NCT04722003, NCT04350138
Nanocage (ferritin)	Influenza	NIAID	1	NCT04579250
Nanocage (ferritin)	Epstein-Barr Virus (EBV)	NIAID	1	NCT04645147
Nanocage (ferritin)	SARS-CoV-2	U.S. Army	1	NCT04784767

Here, we provide an overview of biologically derived nanoparticles, their applications in vaccine development, and their advantages over conventional vaccine platforms. We also discuss how nanoparticle properties, such as size, affects uptake and trafficking by the immune system, and the formulation of biological nanoparticles particles into higher order constructs for controlled release and modulation of the immune response.

## Antigen Uptake and Trafficking to the Lymphatic System

**FIGURE 3:**
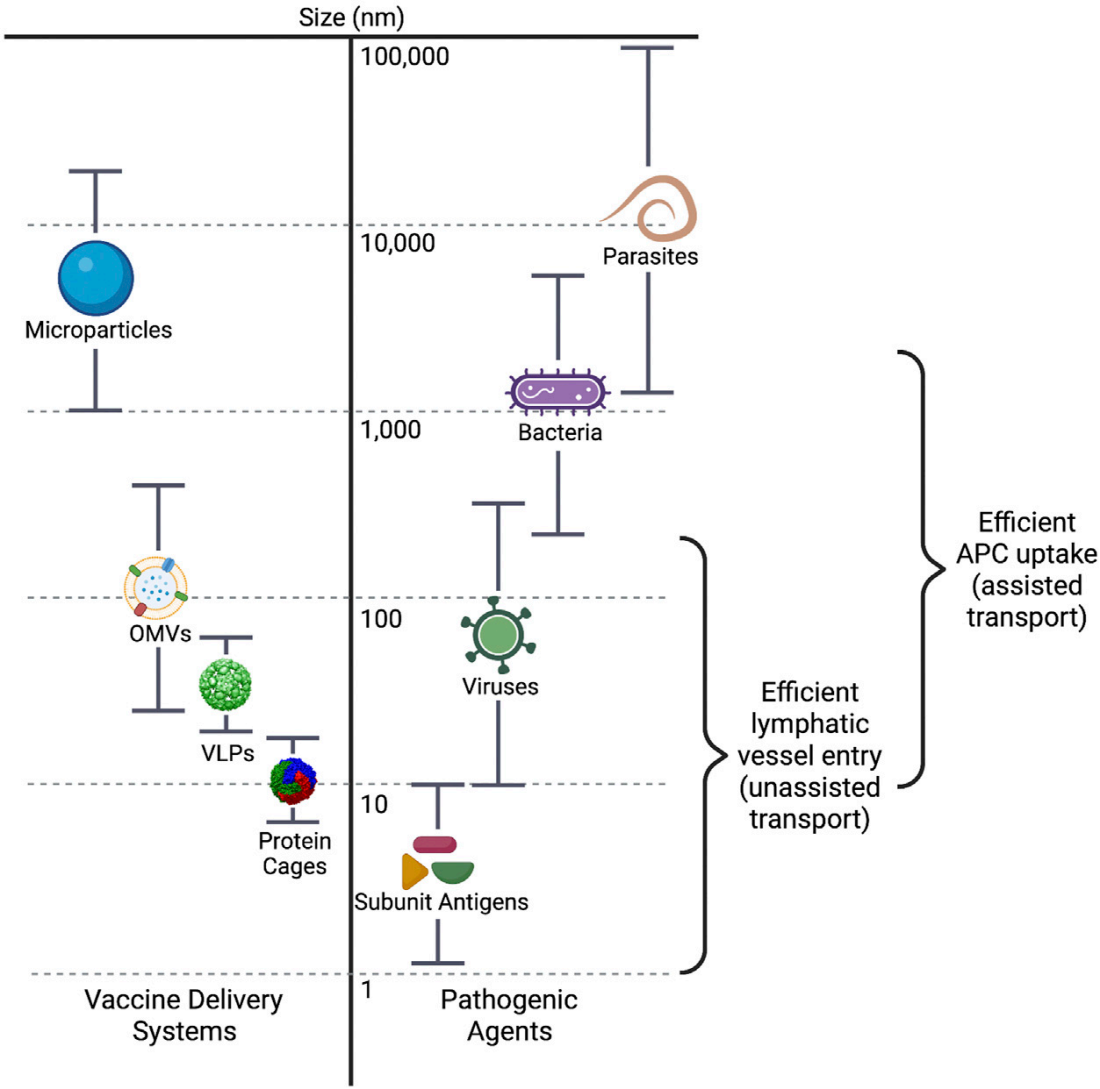
Size ranges of vaccine delivery systems and pathogenic agents. The dimensions of the main particle systems discussed in this review, including microparticle/hybrid structures, are compared to those of pathogenic agents in nanometers. The size ranges for efficient entry into the lymphatic vessels and efficient uptake by antigen presenting cells (APCs) are also indicated. VLPs, virus-like particles; OMVs, outer membrane vesicles.

Antigen uptake and processing, as well as trafficking and lymph node localization, are highly dependent on antigen size, shape, and charge. The sizes of vaccine antigens varies greatly, from subunit antigens that are less than 10 nm in size, to biological or synthetic nanoparticle systems that range from 20 to 200 nm, to whole cell vaccines that can be up to 20 µm in size (Figure 3). Antigen size has significant impact on their uptake by antigen presenting cells (APCs). Antigens with larger surface areas, such as nano- or microparticles and whole-pathogen vaccines, are better able to interact with APCs due to their variety of surface properties such as charge, hydrophobicity, and potential for receptor interaction (Bachmann and Jennings, 2010). Small protein antigens, on the other hand, are inefficiently taken up, and presented by APCs.

Transport of antigen to the secondary lymphoid organs (i.e., lymph nodes, spleen) is required for induction of the adaptive immune response, and size is an important component of how the antigen is transported. Particles that are 20–200 nm may efficiently enter the initial lymphatic vessels (Manolova et al., 2008), where the endothelial cell junctions lining the vessels are leaky, allowing flow through of larger molecules (Singh, 2021). The lymphatic capillaries closer to the lymph node, however, are a much tighter fit, only allowing particles smaller than ∼5 nm to continue forward (Carrasco and Batista, 2007) (Carrasco and Batista, 2007; Singh, 2021). In fact, these smaller particulate antigens, despite not being well taken up by APCs, can be directly transported through specialized small antigen conduits directly to the lymph node (Roozendaal et al., 2009; Fries et al., 2021). Particles in the range of 200–500 nm are unable to enter the lymphatic vessels without assistance. These larger particles are most often taken up by dendritic cells (DCs) and carried into the lymphatic system (Foged et al., 2005; Fries et al., 2021). Interestingly, antigen particles smaller than 200 nm can reach the secondary lymphoid organs within hours of vaccine administration, but transport of larger particles via the DCs can take approximately 24 h (Manolova et al., 2008; Cifuentes-Rius et al., 2021). As antigens with dimensions of 20–200 nm may freely drain to the lymphatic capillaries, designing vaccines within this size range is imperative for facilitation of direct interaction with B cells in the secondary lymphoid organs, and thus activation of a potent immune response (Ding et al., 2021; Singh, 2021; Zinkhan et al., 2021).

Antigen retention by dendritic cells is also affected by nanoparticle size. Zhang et al. recently showed that dendritic cells clear smaller synthetic particles (5–15 nm) from the lymph node follicles very rapidly (within ∼48 h), whereas larger nanoparticles (50–100 nm) were retained for over 5 weeks, allowing the larger particles to present more antigen over time, resulting in a 5-fold greater immune response (Zhang et al., 2000). These data indicate that there is a preferential size range for particle uptake and transport by the immune system, with approximately 50–500 nm being the minimum and maximum dimensions, respectively.

Particle shape does not appear to play a significant role in localization to and activation of B-cells, but certain shapes are preferentially taken up over others by cells of the peripheral immune system. For example, primary mouse bone marrow-derived dendritic cells (BMDCs) preferentially internalize hydrogel nanodiscs over nanorods, indicating that there are unique, geometry-dependent uptake mechanisms taking place (Agarwal et al., 2013; Baranov et al., 2021). In another study, four different gold nanoparticle shapes and sizes (20 nm spherical, 40 nm spherical, 40 × 10 nm rod, and 40 × 40 × 40 nm cubic) were coated with antigens for West Nile Virus (WNV) (Niikura et al., 2013). All of these particles generated WNV-specific antibodies, whereas when WNV protein without AuNPs was administered, the results were similar to the PBS control. The 40 nm spherical nanoparticles were the most successful in generating a protective immune response, resulting in twice as many WNV-specific antibodies as the rod-shaped nanoparticles. These data indicate that the gold nanoparticles not only have an adjuvant effect, but that the adjuvant effect is shape and size dependent. Interestingly, the rod-shaped nanoparticles were internalized to a much greater extent than the other two types, indicating that antibody production does not directly relate to cellular uptake efficiency.

Surface charge is also known to influence particle uptake by immune cells, but there is some debate over whether a positive or negative surface charge better facilitates access to the lymph node. Some studies speculate that improved drainage of negatively charged particles to the lymph node is driven by repulsion between the particles and the negatively charged extracellular matrix (Mueller et al., 2015), while others indicate that they more effectively avoid uptake by cells, and therefore can drain to the lymph node more efficiently (Coffman et al., 2018). Positively charged particles, on the other hand, tend to attract and be internalized by peripheral immune cells at the site of administration, resulting in an enhanced immune response (Fromen et al., 2015; Dacoba et al., 2017).

**FIGURE 4:**
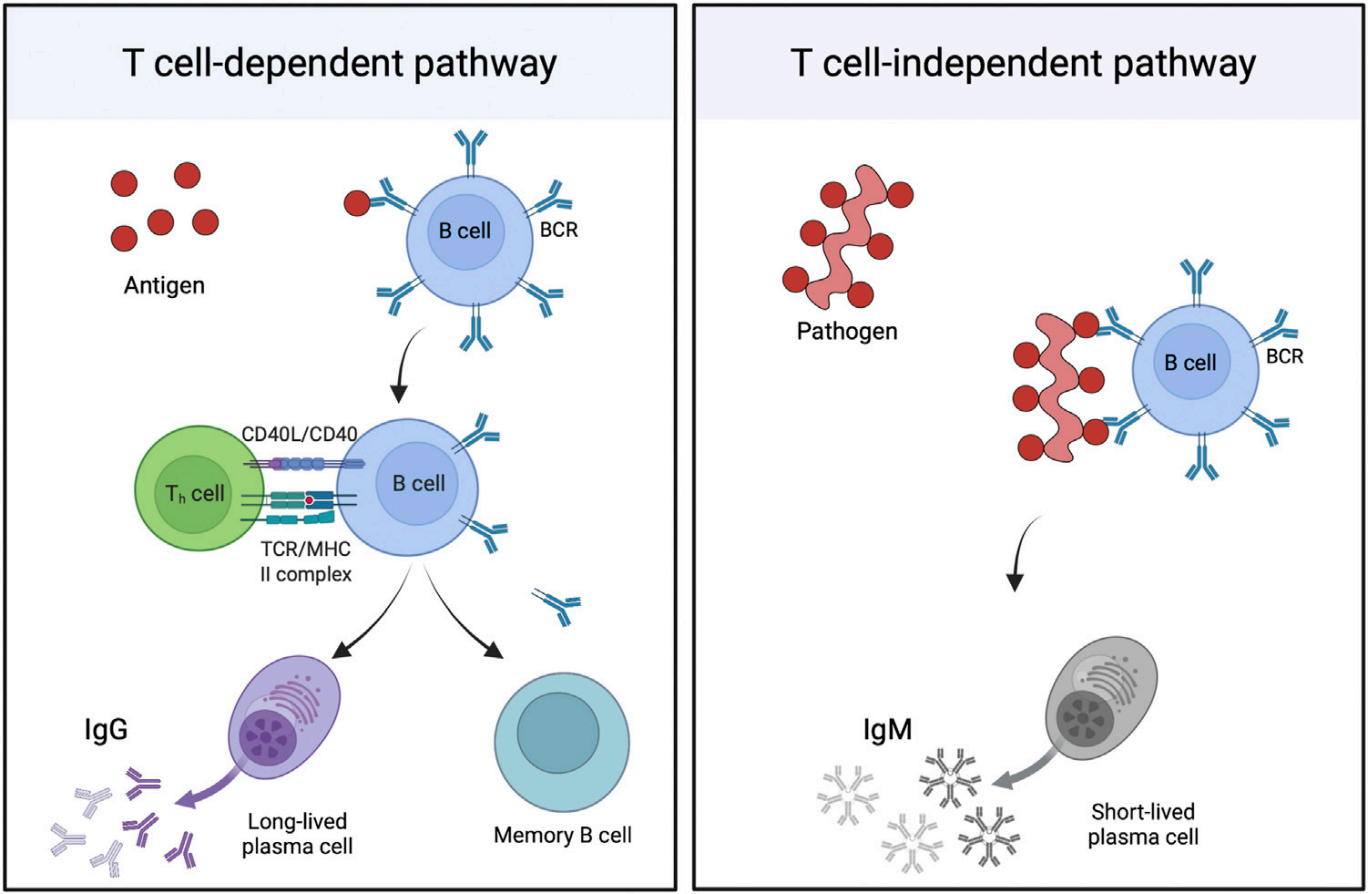
B cell activation. B cells can be activated primarily through two ways: the T cell-dependent (TD), or the T cell-independent (TI) pathway. The TD pathway (left) involves the binding of antigen to B cell receptors (BCRs) on the surface of B cells, internalization and digestion, then display of the resulting peptide on the B cell surface on major histocompatibility complex (MHC) II molecules to CD4^+^ T helper cells. Antigen-specific T cells will then bind to the MHC using their T cell receptor (TCR). The CD40L and CD40 proteins on the T cell and B cell, respectively, will also bind, resulting in activation, proliferation, and maturation of the B cell into memory B cells and long-lived plasma cells, which produce IgG antibodies. The TI pathway (right) is activated by repetitive epitopes on the surface of a pathogen, which heavily crosslink the BCRs on the surface of the B cell. This most often results in B cell activation, maturation, and the generation of short-lived plasma cells, which primarily produce IgM. This figure was generated using Biorender.com.

Once the antigen is within the lymph node, it can activate B cells in two different ways (Figure 4). T cell dependent activation involves binding of antigen to the B cell receptor (BCR) on B cells, internalization and degradation of the antigen, and presentation of peptides on major histocompatibility complex (MHC) II molecules to CD4^+^ T helper cells. Following T cell receptor binding to the MHC II molecules and release of co-stimulatory molecules, B cells become activated, forming germinal centers in the follicular region of lymph nodes, and beginning proliferation. These cells then undergo affinity maturation and class switching, guided by T helper cells, which results in development of high affinity IgG antibody producing plasma cells and long-lived memory B cells. Alternatively, B cells can bind directly to PAMPs or through BCRs and extensively crosslink to repeated epitopes on the pathogen surface, resulting in proliferation of B cells, their differentiation into plasma cells, and production of IgM antibodies. This T cell independent response, while useful in the short-term, does not generally result in production of memory B-cells and long-lived plasma cells (Chaplin, 2010; Cyster and Allen, 2019).

Lymphocytes require additional signals to promote maturation and appropriate antibody class switching. These signals are usually provided by the antigen presenting cell, which is activated to increase release of pro-inflammatory cytokines (especially interleukins (IL), such as IL-10, IL-12, and IL-23, etc.) in response to stimulatory signals from the pathogen. These signals correspond with activation of pattern recognition receptors, such as TLRs and NLRs, and which recognize specific PAMPs. In many cases, synthetic adjuvants, such as aluminum hydroxide, induce these signals, but biologically derived nanoparticles naturally contain many PAMPs that can be utilized to activate the immune system without the need for additional adjuvant (Zhang et al., 2000; Li Y. et al., 2021).

## Virus-Like Particles

Virus-like particles (VLPs), a type of subunit vaccine, are formed from the self-assembly of viral capsid proteins into particles that mimic the parent virus but are incapable of replication or infection. The inherent inability of VLPs to infect or replicate alleviates potential vaccine risks associated with live-attenuated or inactivated vaccines, such as spontaneous reversion to a pathogenic phenotype or incomplete inactivation. These characteristics impart VLPs with a favorable safety profile and enable low-containment manufacturing. Furthermore, many VLPs contain highly repetitive, dense (50–100 Å spacing), and rigid structures on their surface, allowing them to naturally crosslink B cell receptors. This effect, despite being T cell-independent, then leads to strong stimulation of B cells and induction of a robust and long-lasting antibody response (Bachmann et al., 1993; Fries et al., 2021) that can even be achieved without the addition of synthetic adjuvants (Zhang et al., 2000; Li Y. et al., 2021). Furthermore, VLPs naturally encapsulate bacterial nucleic acids when propagated in bacterial expression systems, which further activate antigen-presenting cells and B cells (Mohsen et al., 2021). The success of the VLP platform has already resulted in many FDA-approved VLP-based vaccines, including Cervarix^®^ (Harper et al., 2004) and Gardasil^®^ (Villa et al., 2005) for Human Papilloma Virus (HPV), and Engerix^®^ (Keating et al., 2003) and Recombivax^®^ (Van Damme et al., 2009) for Hepatitis B Virus (HBV) (see Table 1).

A variety of viruses are used as VLP vaccine platforms, including bacteriophage, insect viruses, and plant viruses (Table 3). Single-stranded RNA viruses, such as MS2 bacteriophage, AP205, and Qβ, are widely used VLP platforms, as their small genomes can be easily modified to generate new antigens on their surfaces (Yadav et al., 2021). In one study, MS2 coat proteins were fused with the minor capsid protein (L2) of HPV to generate MS2-L2 VLPs (Zhai et al., 2017). These vaccines were then administered to mice and challenged against various HPV pseudovirus types (Zhai et al., 2017, 2019; Yadav et al., 2021). The MS2-L2 VLPs were protective against all tested viral strains, and one study even confirmed that the protective antibodies generated from vaccination with these VLPs last for over 9 months (Yadav et al., 2021). Bacteriophage T4 have also been developed as a vaccine platform, specifically for use against influenza (Li M. et al., 2021). In addition to bacteriophage, insect and plant viruses may also be genetically modified to generate chimeric viruses. Chimeric viruses contain genetic material from two different viruses, which results in fusion proteins that incorporate epitopes from both viruses. Recombinant chimeric VLP vaccines have been developed for Chikungunya virus (CHIKV), based on the insect-specific alphavirus, Eilat virus (EILV) (Erasmus et al., 2017); *Bacillus anthracis*, based on cowpea mosaic virus (CPMV) (Phelps et al., 2007) and Flock house virus (Venter et al., 2011); SARS-CoV-2 and MERS, based on cucumber mosaic virus (CMV) (Mohsen et al., 2021, 2022); and SARS-CoV-2 and *Yersinia pestis*, based on tobacco mosaic virus (TMV) (Arnaboldi et al., 2016; Royal et al., 2021).

**TABLE 3:** VLPs used as vaccine platforms.

VLP platform	Pathogen targeted	Reference
Bacteriophage		
MS2	HPV	Zhai et al. (2019), Yadav et al. (2021)
AP205	*Escherichia Coli*	Govasli et al. (2019)
	HPV and Malaria	Janitzek et al. (2019)
	Malaria	Yenkoidiok-Douti et al. (2019)
	Influenza	Thrane et al. (2020)
	SARS-CoV-2	Fougeroux et al. (2021), Liu et al., (2021)
Qβ	Zika	Basu et al. (2018)
	Chikungunya	Basu et al. (2020)
	Dengue	Warner and Frietze, (2021)
	SARS-CoV-2	Ortega-Rivera et al. (2021)
T4	Influenza	Li et al. (2021a)
Plant Virus		
Tobacco Mosaic Virus	*Yersinia pestis*	Arnaboldi et al. (2016)
	SARS-CoV-2	Royal et al. (2021)
Cowpea Mosaic Virus	Anthrax	Phelps et al. (2007)
Cucumber Mosaic Virus	Zika	Cabral-Miranda et al. (2019)
	SARS-CoV-2	Zha et al. (2021), Mohsen et al. (2022)
	MERS	Mohsen et al. (2021)
Papaya Mosaic Virus	HPV	Thérien et al. (2017), Laliberté-Gagné et al. (2019)
Insect Virus		
Flock House Virus	Anthrax	Venter et al. (2011)
Eilat Virus	Chikungunya	Erasmus et al. (2017)

**FIGURE 5:**
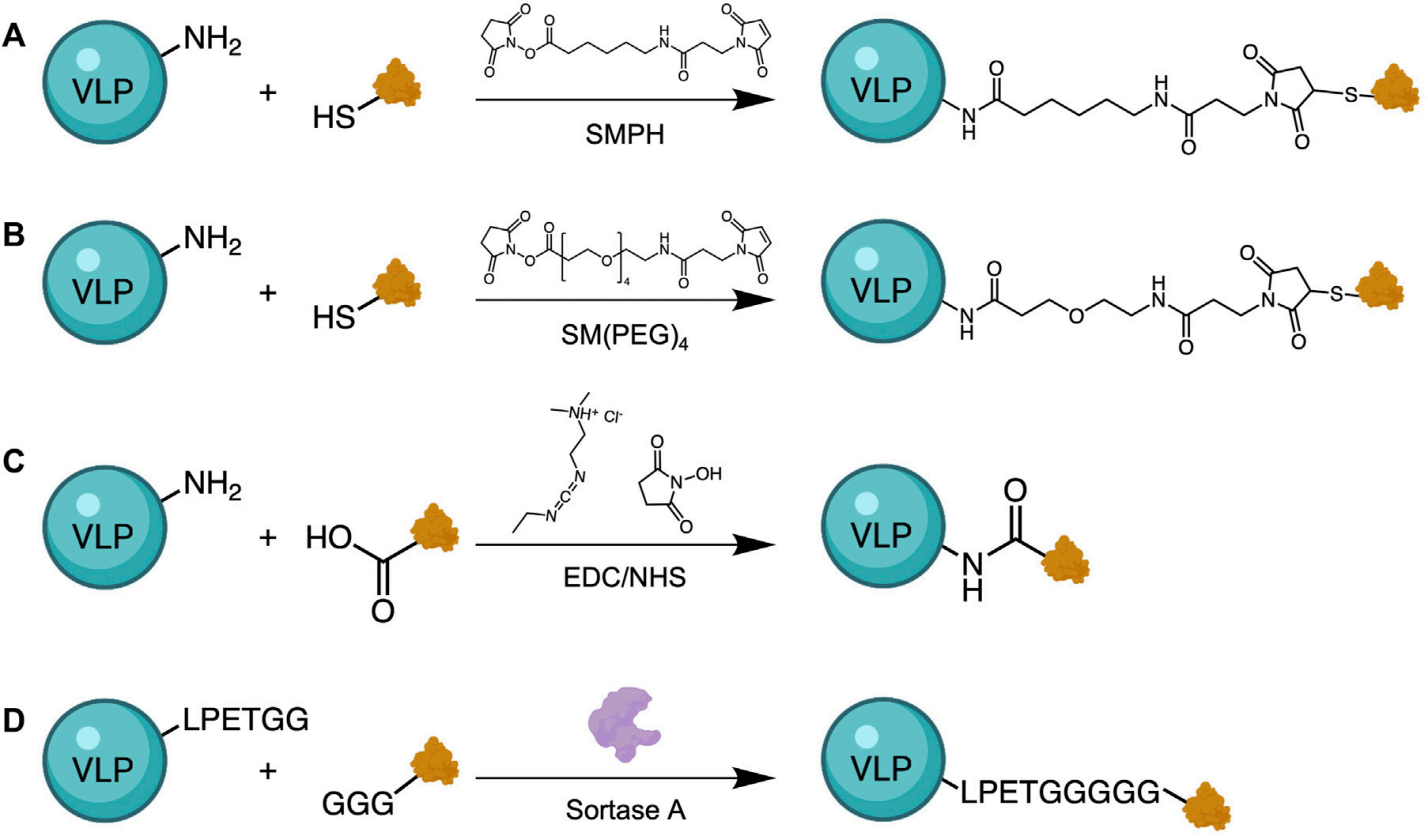
Approaches for antigen conjugation to VLPs. Heterobifunctional crosslinkers, such as SMPH **(A)** or SM(PEG)_4_ **(B)**, have been used to link VLPs with an antigen of interest. These linkers react with free primary amines from lysine residues on VLPs and thiols from genetically inserted cysteine residues on the antigen. Alternative methods use carbodiimide chemistry to activate the less reactive carboxylic acid residues on the antigen for reaction with amines on the VLPs **(C)**, and Sortase-mediated transpeptidation **(D)**. Sortase A catalyzes the binding of a target sequence (GGG), often bound to antigen, with an LPETGG tag genetically added to the VLP surface.

Direct chemical conjugation of antigen to VLPs is another strategy for the development of VLP-based vaccines. When genetic insertion of Zika virus (ZIKV) epitopes into MS2 and PP7 bacteriophage genomes was unsuccessful, Basu et al. covalently conjugated the epitopes to Qβ via a heterobifunctional crosslinker, SMPH (succinimidyl 6-((beta-maleimidopropionamido) hexanoate)) (Figure 5) (Basu et al., 2018). ZIKV epitopes were modified to contain a cysteine residue containing a free thiol group for conjugation, while the amine groups on surface-exposed lysine residues of Qβ were reacted with the other end of the linker. When tested in a mouse model, immunized mice were not protected from Zika challenge, but serum antibodies from immunized mice neutralized the virus *in vitro*, indicating that this approach has potential for vaccine development. A similar strategy was also used to conjugate Qβ VLPs to CHIKV epitopes (Basu et al., 2020) and Dengue virus (DENV) epitopes (Warner and Frietze, 2021), as well as to conjugate CMV VLPs to recombinant SARS-CoV-2 spike protein (Zha et al., 2021) and ZIKV immunogens (Cabral-Miranda et al., 2019). Other linkers used for conjugation of VLP platforms to pathogenic antigens include SM(PEG)_4_ (N-hydroxysuccinimide-poly (ethylene glycol)_4_-maleimide) (Ortega-Rivera et al., 2021) for conjugation of Qβ to SARS-CoV-2 spike protein, and carbodiimide chemistry for conjugation of *Yersenia pestis* virulence factors (Arnaboldi et al., 2016) to TMV VLPs. Another interesting method that others have used to attach the VLP carrier to the antigen of interest is through the use of sortase-mediated antigen coupling (Thérien et al., 2017; Laliberté-Gagné et al., 2019). Sortase-mediated conjugation allows for direct attachment of various compounds that contain the target sequence (multiple glycine residues) to VLPs that have been modified to express a specific amino acid tag (LPETGG), without covalent conjugation (Figure 5D). This universal approach allows the attachment of virtually any antigen or adjuvant as long as they contain the tag sequence.

Most vaccine platforms have focused on the use of nonenveloped VLPs, but lipid enveloped VLPs (eVLPs) have also been studied, mainly those derived from the baculovirus expression system in insect cells (López-Macías et al., 2011; Dai et al., 2018b; Hu et al., 2021), or in plant systems (D’Aoust et al., 2008; Landry et al., 2010; Pillet et al., 2016). Nonenveloped VLPs are much less complex than eVLPs, and can be produced in prokaryotic systems, making them easily scalable, cost-effective, and rapid to manufacture. The presence of a lipid bilayer in eVLPs necessitates the use of eukaryotic hosts for expression, which increases the overall production time and cost. Additionally, eVLPs are much more sensitive to their external environment, such as temperature and shear forces, which may destroy the particle integrity. On the other hand, eVLPs can express membrane-bound antigens and, as they use eukaryotic hosts, can also incorporate post-translational modifications, giving them some advantages over their simpler counterparts (Dai et al., 2018a). In a recent study by Hu et al., three recombinant insect-derived influenza eVLPs, each expressing different influenza antigens, were tested for their ability to promote a protective immune response (Hu et al., 2021). The different eVLPs were mixed, then injected intramuscularly into mice, and chickens. This resulted in a robust antibody response and complete protection against lethal influenza virus challenge in both animal models. Although the vaccines administered to the chickens contained adjuvant, the vaccines administered to the mice did not, indicating that a protective response may still be elicited by eVLPs alone. Plant-derived eVLPs have been met with success in clinical trials. The Canadian company Medicago recently completed Phase 3 clinical trials with their quadrivalent eVLP influenza vaccine derived from plant cells (NCT03301051), establishing their platform as a viable alternative to current influenza vaccines on the market (D’Aoust et al., 2008; Ward et al., 2020).

## Outer Membrane Vesicles

**FIGURE 6:**
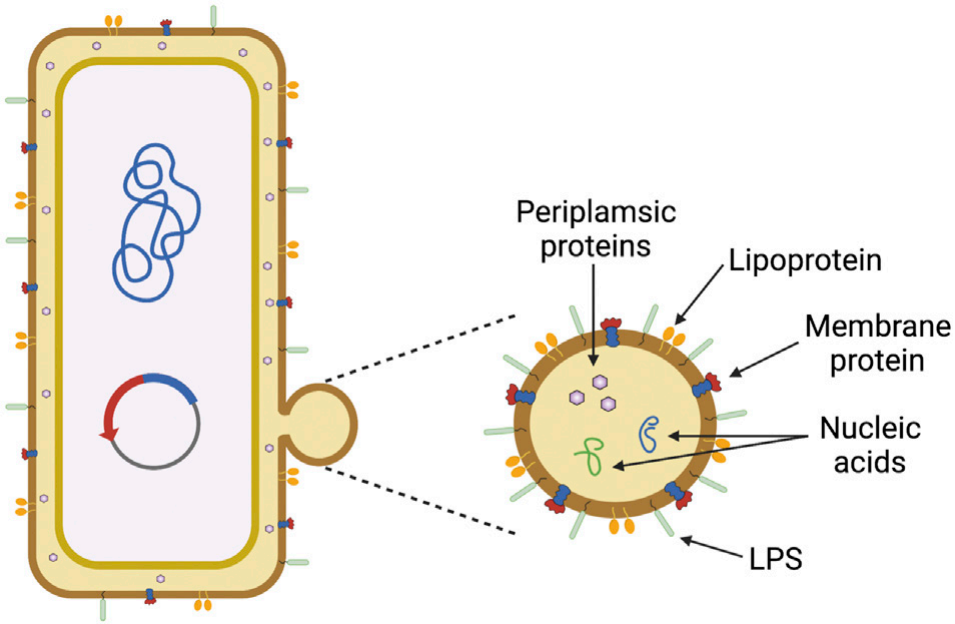
Outer membrane vesicles (OMVs) and pathogen-associated molecular patterns (PAMPs). OMVs naturally bud from the bacterial outer membrane (left), which allows them to display many bacterially-derived PAMPs (right) that activate toll-like receptors (TLRs) to enhance the memory response. This figure was generated using Biorender.com.

While VLPs can express heterologous antigens on their surface from various types of pathogens, they often lack many bacterially derived PAMPs. Outer membrane vesicles (OMVs) naturally bud from the outer membrane of Gram-negative bacteria, although studies have shown that Gram-positive bacteria and archaea produce similar extracellular vesicles (Liu et al., 2018; Gill et al., 2019). OMVs contain many PAMPs associated with bacterial outer membranes, including lipopolysaccharide (LPS), periplasmic components, nucleic acids, lipoproteins, and other outer membrane proteins (Figure 6). In this way, OMVs are often considered “self-adjuvanting,” that is, the PAMPs contained both on and within the OMVs provide an enhanced immune response to any antigenic compounds being carried. The clinical capabilities of the OMV platform have previously been established, as there are currently two licensed vaccines on the market containing OMVs, also known as outer membrane protein complexes (OMPC). Bexero^®^, a Meningitis B (MenB) vaccine created by GlaxoSmithKline, contains OMVs as an artifact of the production process (Giuliani et al., 2006). PedVaxHIB^®^ is a conjugate vaccine that uses the polysaccharide polyribosylribitol phosphate (PRP) of *Haemophilus influenzae* type b (Hib) covalently bound to an OMPC of *Neisseria meningitidis* (Vella et al., 1990) (see Table 1).

**FIGURE 7:**
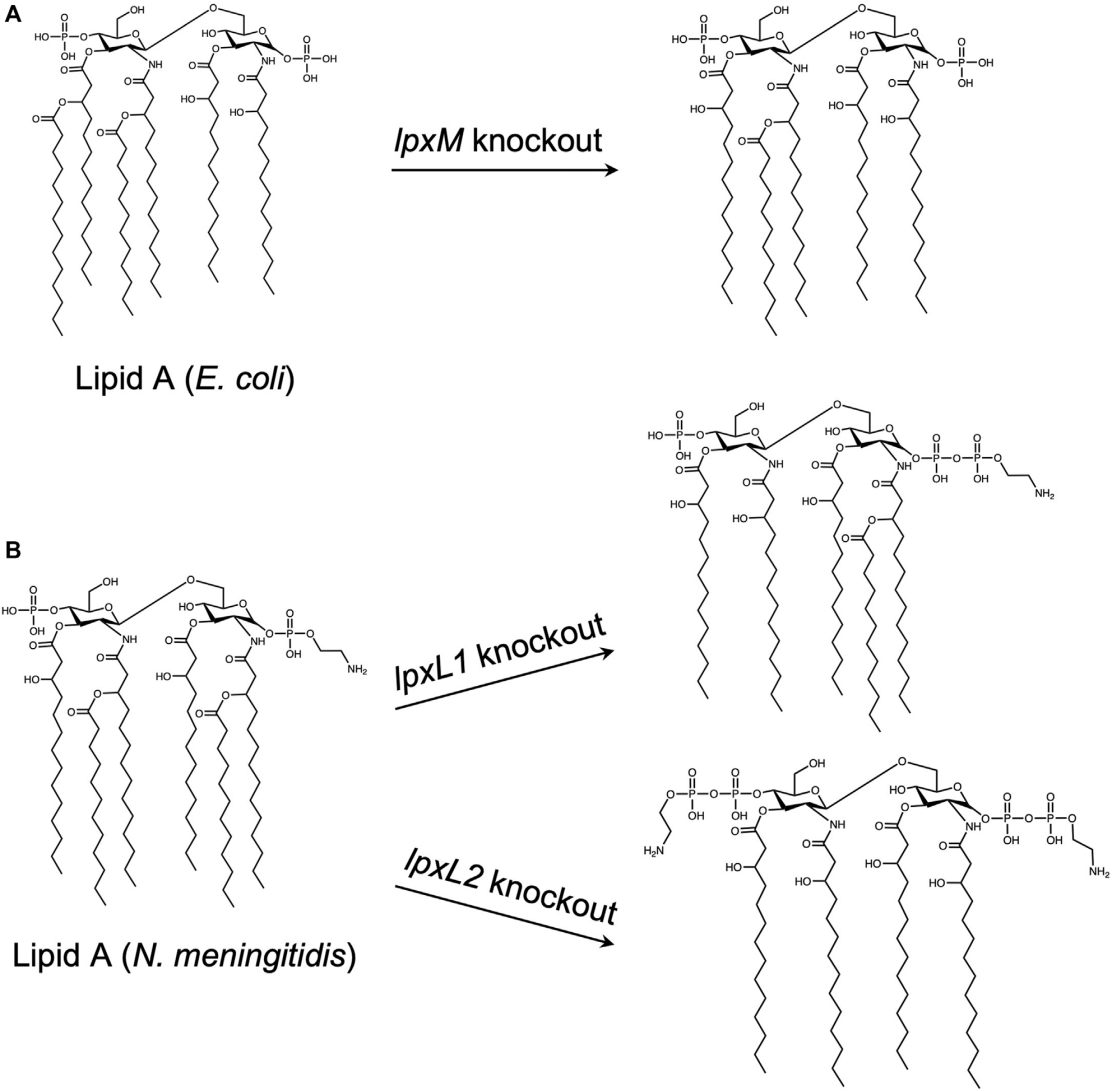
Effect of gene knockouts on the structure of Lipid A. The lipid A domain of lipopolysaccharide (LPS) is mainly responsible for the toxicity associated with gram negative bacteria. As outer membrane vesicles (OMVs) natively contain LPS, and LPS is necessary for membrane stabilization, many studies have focused on detoxifying LPS by modifying the structure of lipid A, rather than removing it entirely. Both *E. coli* and *N. meningitidis* lipid A structures are natively hexa-acylated. Knockout of *lpxM* in *E. coli* **(A)** and *lpxL1* in *N. meningitidis* **(B)**, (top) results in a penta-acylated lipid A, whereas *lpxL2* knockout in *N. meningitidis* **(B)**,(bottom) results in a tetra-acylated species. (Van Der Ley et al., 2001; Mamat et al., 2015; Chen et al., 2016).

Although OMVs are replication deficient and are not infectious, resulting in an increased safety profile over whole-pathogen vaccines, they may also contain high amounts of LPS in their membranes. LPS, or endotoxin, is pyrogenic and highly toxic, and can lead to fever, uncontrolled inflammation, and sepsis (Opal, 2010). In one study, after mice were immunized with OMVs, some experienced over 20% weight loss, and were subsequently euthanized. This result was attributed to a reaction to LPS present on the OMVs (Kuipers et al., 2015). Depending on the way OMVs are produced, the amount of endotoxin may be reduced, but not fully removed. Detergents are often be used to extract LPS, but this method may impact the immunomodulatory effects of the OMVs by removing other PAMPs needed to generate a protective immune response (van de Waterbeemd et al., 2010). As an alternative, some groups have genetically modified the structure of the lipid A component of LPS to obtain bacterial strains capable of producing endotoxin-free OMVs (van der Ley et al., 2001; Rosenthal et al., 2014; Chen et al., 2016). The effect on the adjuvanticity, however, may vary depending on the mutation. In one case, where the *lpxM* gene was knocked out in *E. coli*, there was no significance difference in serum antibody titers and survival upon challenge with OMV vaccines containing native or modified LPS, suggesting that OMVs with modified LPS may provide the same level of protection as native OMVs (Chen et al., 2016). In another study, however, insertional inactivation of *lpxL1 N. meningitidis* resulted in retainment of adjuvant activity and reduced toxicity, but inactivation of *lpxL2* resulted in both reduced adjuvant activity and toxicity, indicating that the type and location of LPS mutation must be considered carefully (van der Ley et al., 2001). The effect of these knockouts on the structure of lipid A is shown in Figure 7.

OMV-based vaccines can be made directly from the target pathogen or made recombinantly via genetic engineering of safer bacterial hosts. Collecting and purifying OMVs from pathogenic microbes requires greater attention to purification techniques and reduces safety compared to genetic engineering of nonpathogenic bacteria. Bacterial engineering is also amenable to the use of endotoxin-free bacteria and allows expression of foreign antigens on or within the OMV. There is evidence that antigen presentation on the exterior of the OMV promotes a superior immune response in comparison to antigen encapsulated within the OMV (Hess et al., 1996; Kang and Curtiss, 2003; Barat et al., 2012; Salverda et al., 2016). The presentation of antigen on the surface of OMVs presents its own level of difficulty, because most proteins do not naturally localize to the outer membrane. Therefore, antigen fusion with outer membrane proteins or autotransporters, such as cytolysin A (ClyA) (Chen et al., 2010; Rappazzo et al., 2016), Sec-dependent signal peptide (spPelB) (Weyant et al., 2021), and hemoglobin protease (Daleke-Schermerhorn et al., 2014), is commonly used to display antigen on the OMV outer surface. Chemical conjugation of the antigen to the exterior of the OMV can also be used to present antigen (or additional adjuvant) on the surface of the OMV, as was done for the commercially available vaccine PedVaxHIB^®^. This can be accomplished through conjugation with antigen through amine groups or free thiols, as discussed for VLPs (Wu et al., 2006; Scaria et al., 2019; Micoli et al., 2020).

**FIGURE 8:**
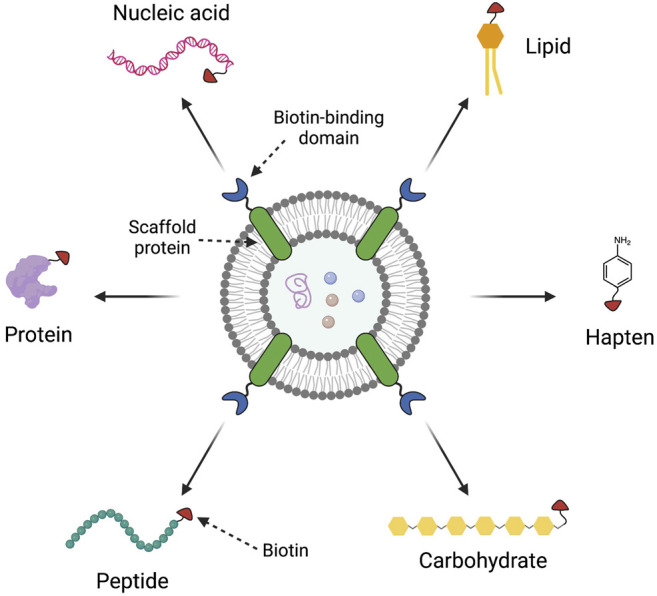
The Addvax approach for external modification of outer membrane vesicles (OMVs). The Addvax approach, developed by Weyant et al. (2021), consists of a scaffold protein inserted into the OMV membrane that is fused to a biotin-binding domain, such as avidin. These avidin-decorated OMVs can then be reacted with any biotinylated compound, including proteins, peptides, carbohydrates, haptens, lipids, and nucleic acids, for conjugation to the OMV. This figure was generated using Biorender.com.

As mentioned, OMV-based vaccines currently exist for the prevention of Hib and MenB infections. Recent literature has focused on the development of OMV vaccines for influenza (Rappazzo et al., 2016; Watkins et al., 2017b), malaria (Scaria et al., 2019), pertussis (Raeven et al., 2020; Carriquiriborde et al., 2021), Lyme disease (Klouwens et al., 2021), plague (Carvalho et al., 2019; Wang X. et al., 2020), and SARS-Cov-2 (Gaspar et al., 2021; Thapa et al., 2021; van der Ley et al., 2021). Due to the inherent flexibility and capability of the OMV platform, there is interest in expressing heterologous antigens from different pathogens on the same OMV to create novel combined vaccines (König et al., 2021). One recently reported strategy is a clever universal approach termed “Addvax,” avidin-based dock-and-display for vaccine antigen cross-linking (Weyant et al., 2021). The Addvax approach utilizes an avidin binding moiety fused with an outer membrane protein so that the biotin binding domain may be expressed on the OMV exterior (Figure 8). This allows any compound that can be biotinylated to be conjugated to the OMV surface. Through this mechanism, OMVs have been decorated with a diverse array of compounds, ranging from proteins and glycans to lipids and peptides. This platform could be conceivably used to decorate the same OMV with different antigens, depending on the reaction ratios used, and steric hindrance between the different antigens.

## Protein Nanocages

While viruses and VLPs are often classified as nanocages, this section considers cages made from non-viral protein subunits, rather than those derived from a parental pathogen. Nanocages, similar to VLPs, may also be considered protein subunit vaccines. Protein nanocages self-assemble from a small number of subunits to form symmetrical, macromolecular containers with a vast diversity in shape and size (Flenniken et al., 2009; Chakraborti and Chakrabarti, 2019). These characteristics often allow them to mimic native pathogens, as they contain high density repetitive regions on their surfaces that may be recognized and crosslinked by B-cell receptors. In recent years, ferritin-based nanocages have emerged as a promising platform for vaccine development.

**FIGURE 9:**
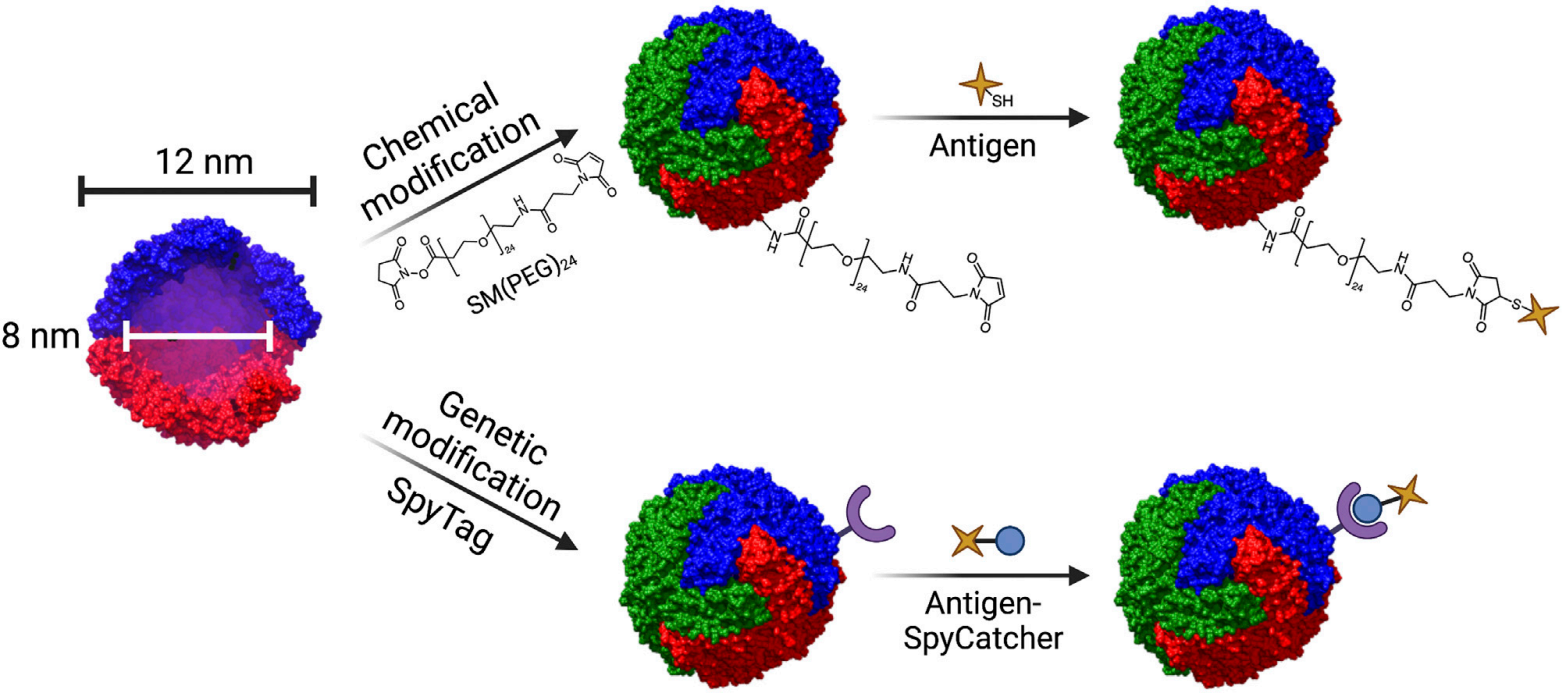
Structure of the ferritin nanocage and techniques used for conjugation of antigen. Ferritin nanocages have an outer diameter of 12 nm, and a hollow inner core of 8 nm in diameter. Ferritin can be modified in a variety of ways to conjugate antigen. Chemical modification involving heterobifunctional linkers such SM(PEG)_24_ enable conjugation of antigen via click chemistry (top). Genetic modification can enable conjugation of antigen through the SpyTag/SpyCatcher system (bottom). The crystal structure of L-ferritin (pdb id. 2fg8 (Wang et al., 2006)) was used to model the ferritin nanocage. The nanocage was constructed using UCSF Chimera, developed by the Resource for Biocomputing, Visualization, and Informatics at the University of California, San Francisco, with support from NIH P41-GM103311 (Pettersen et al., 2004).

Ferritin, most often derived from *Helicobacter pylori*, is composed of 24 subunits that self-assemble into a 12 nm diameter spherical cage with a hollow core. It is used in humans to store iron (Chakraborti and Chakrabarti, 2019). Target antigens may be introduced to the surface of ferritin cages by either genetic modification or chemical conjugation (Figure 9). Genetic modification of ferritin not only permits antigenic display, but also allows for the introduction of mutations that may improve functionality, such as addition of a cysteine group to the exterior to allow for conjugation of adjuvants via click chemistry (Kamp et al., 2020). Due to its versatility, ferritin has been engineered as a vaccine platform to display antigens from various pathogens, including *Borrelia burgdorferi* (Kamp et al., 2020), influenza (Kanekiyo et al., 2013; Yassine et al., 2015; Qi et al., 2018; Wei et al., 2020), Epstein-Barr virus (Kanekiyo et al., 2015; Qu et al., 2021b, 2021a), hepatitis B (Wang W. et al., 2020), rotavirus (Li et al., 2019), and, most recently, SARS-Cov-2 (Joyce et al., 2021; Kalathiya et al., 2021; Kang et al., 2021; King et al., 2021; Wuertz et al., 2021).

Ferritin nanocages have shown impressive correlates of protection in animal studies. For example, when a recombinant influenza vaccine was made using a hemagglutinin-stem immunogen fused to ferritin, vaccination of mice and ferrets generated a robust antibody response that fully protected mice and partially protected ferrets against viral challenge (Yassine et al., 2015). Furthermore, passive immunization of unvaccinated mice with serum antibodies from vaccinated mice also conferred protection against lethal infection. In another study, non-adjuvanted ferritin-M2e cages were used to vaccinate mice intranasally (Qi et al., 2018). The results of this study showed that, even without adjuvant, the ferritin nanocage vaccines elicited production of M2e-specific IgG antibodies, secretion of IgG antibodies, and a strong T cell response. Even more impressively, the non-adjuvanted intranasal vaccine conferred complete protection against viral challenge, demonstrating the further potential of ferritin nanocages as a needle-free vaccine platform.

As mentioned above, in addition to influenza, ferritin nanocages have also been studied for vaccines against Lyme disease. Kamp et al. fused ferritin to the outer surface protein A (OspA) of *Borrelia* bacteria, the causative agents of Lyme borreliosis (Kamp et al., 2020). After ferritin-OspA nanoparticles self-assembled, the squalene-based adjuvant AF03 was added, and the vaccine was administered to mice and non-human primates via intramuscular injection. The nanoparticles not only generated high antibody titers against seven different strains of *Borrelia* in both animal models, but these titers were higher than those of a licensed non-human Lyme disease vaccine, and the response lasted for over 6 months. When chemically conjugated to a TLR7/8 agonist, the ferritin-OspA vaccine also protected against two tick-fed murine challenge models, indicating that surface modification does not affect potency of the immune response. While adjuvant was mixed with each vaccine before administration, the ferritin-OspA platform still shows promise as a protective vaccine against Lyme disease.

Various ferritin-based vaccines are in clinical trials, with two influenza trials recently completing Phase I (NCT03186781, NCT03814720), and trials for Epstein-Barr virus (NCT04645147), SARS-Cov-2 (NCT04784767), and a third influenza trial (NCT04579250) currently ongoing (see Table 2). In addition to ferritin, other proteins naturally undergo self-assembly into cage-like structures, such as luzamine synthase (Ra et al., 2014; Ladenstein and Morgunova, 2020), the E2 subunit of pyruvate dehydrogenase (Molino et al., 2017), and small heat shock protein (Wang D. et al., 2020; Demchuk and Patel, 2020), but they have not been studied for their ability to generate a protective immune response.

## Hybrid Structures

Biologically-derived particles are clinical successes, as evidenced by their completion of clinical trials and translation into commercially available vaccines. However, similar to other commercially available vaccines, many biologically-derived nanovaccines still require boosters, adjuvants, and multiple doses to promote a protective immune response. Administration and manufacturing costs associated with these additional doses could be reduced with a single dose vaccine. More significantly, in pandemic situations, a vaccine that can induce a protective immune response in only one dose is preferred, minimizing the time needed to complete the vaccine regimen and potentially reducing hospitalizations and deaths. As shown by the currently available COVID-19 vaccines, however, most pandemic-responsive vaccines are not capable of this level of protection.

As discussed previously, biologically-derived nanoparticles have some advantages over classical vaccine platforms. These particles mimic the parent pathogen, contain densely repetitive and rigid surface structures, and are nanoscale in size, all of which help to promote a highly efficacious and protective immune response while maintaining an improved safety profile over whole pathogen vaccines. While studies have confirmed that some types of biologically-derived nanoparticles are capable of inducing a protective immune response in a single dose (Erasmus et al., 2017; Hu et al., 2021; Joyce et al., 2021; Wuertz et al., 2021)**,** there is evidence that incorporating these nanovaccines into higher-order structures, such as microspheres, microparticles, or hydrogels, may provide a way to better invoke, and control the immune response. Formulation of these nanoparticles into higher order structures may also enable development of effective single-dose vaccines**.** Additionally, selection of an appropriate encapsulation agent may improve potency, increase stability, and enable the use of less stringent storage conditions for biologically-derived nanoparticle vaccines, as it does for protein-based vaccines (Carreño et al., 2016).

Both VLPs and OMVs have been studied for encapsulation and release from larger ordered structures, with the encapsulating agents in more recent years mainly being polymer-based (Watkins et al., 2017a; Jamaledin et al., 2020; Pastor et al., 2020; Gomes et al., 2021), and although liposomes have also been used (Kim et al., 2020) (Table 4).

**TABLE 4:** Hybrid structures used in the formulation of biologically-derived nanoparticles.

Hybrid structure	Formulation	Target pathogen	Reference
Micron-size particles	Recombinant OMV containing M2e antigens derived from Clearcoli^®^ encapsulated in PLGA microparticles	Influenza	Watkins et al. (2017a)
	Porcine circovirus type 2 VLPs encapsulated in chitosan microparticles	Porcine circovirus type 2	Bucarey et al. (2014)
	OMVs isolated from *Neisseria meningitidis* encapsulated in dextran- or mannan-based microspheres	*Neisseria meningitidis*	Arigita et al. (2004)
	Foot-and-mouth disease VLPs encapsulated in liposomes	Foot-and-mouth disease	Kim et al. (2020)
	Bacteriophage-based (f3) VLPs encapsulated in PLGA microparticles	None	Jamaledin et al. (2020)
Polymeric gel	M2e VLPs encapsulated in a polymeric matrix	Influenza	Gomes et al. (2021)
	Lyophilized OMVs derived from heat treated *Shigella flexneri* ΔtolR bacteria, encapsulated in a polymeric matrix	*Shigella flexneri*	Pastor et al. (2020)
Nano-sized particles	Foot-and-mouth disease VLPs complexed with gold nanocages	Foot-and-mouth disease	Teng et al. (2021)
	OMVs isolated from *Bordetella pertussis* encapsulated in sodium alginate nanoparticles	*Bordetella pertussis*	Rami et al. (2021)

One of the first studies to investigate the encapsulation of OMVs, Arigita et al. developed dextran, and mannan microspheres containing meningococcal OMVs expressing neisserial pore protein A (PorA) (Arigita et al., 2004). Interestingly, the serum antibody titers for PorA showed no difference between mice immunized with OMVs alone and OMVs encapsulated in either of the two microspheres, indicating that immunogenicity was not affected by encapsulation. In a more recent study, Watkins et al. investigated encapsulation of M2e-OMVs into PLGA microparticles as a single-dose, long lasting vaccine platform for influenza (Watkins et al., 2017a). Four weeks post vaccination, mice generated serum antibodies against M2e that were equivalent with those generated at 8 weeks by mice immunized in a typical prime/boost vaccination. Furthermore, mice challenged with a lethal dose of influenza virus PR8 (H1N1) both 10 weeks and 6 months post vaccination resulted in 100% survival for both groups of vaccinated mice at both time points. Additionally, mice in both groups showed sustained antibody titers for 6 months post prime vaccination. Taken together, this study supports the potential for a controlled release hybrid platform as a single-dose, long lasting vaccine.

Veterinary vaccines may also benefit from hybrid vaccines that encapsulate biologically-derived nanoparticles. As injectable vaccines are often impractical for mass vaccination of farm animals, oral or inhalable vaccines are preferred, which can be less effective than the injectable formulations. Hybrid formulations may provide the controlled release needed to develop effective oral vaccines. Chitosan microparticle vaccines were used to encapsulate VLPs derived from porcine circovirus type 2 (PCV2), which was administered to mice via oral gavage (Bucarey et al., 2014). The immune response of the orally-administered chitosan-encapsulated PCV2-VLPs was compared to a subcutaneously administered, commercially available PCV2 vaccine. Both vaccines generated similar cytokine and T-cell responses against PCV2, indicating a similar protective response. While this study focuses on VLPs derived from a porcine virus, this experimental hybrid vaccine provides a promising foundation for the future development of oral vaccines in humans.

In addition to microparticle systems, hydrogels also have great applicability to nanoparticle vaccine formulation. Pastor et al. recently developed a thermosensitive hydrogel incorporating lyophilized OMVs for intranasal delivery (Pastor et al., 2020). The copolymer gel was formulated using the polymer Gantrez^®^ AN119 and the surfactant Pluronic^®^ F127. The OMV-loaded hydrogel showed a rapid release profile, releasing the majority of its cargo within 30 min. Interestingly, use of the hydrogel platform prolonged the antigen residence time in the nasal epidermis of intranasally vaccinated mice from 30 min (in mice given free OMVs) to 2 h. However, the immune response generated by these OMV-loaded hydrogels was not evaluated. While the results are promising, this platform must be further investigated for use as a vaccine platform. In addition to OMVs, ferritin has been also incorporated into a polymer microgel (Budiarta et al., 2021). Encapsulation into the microgel was explored as a strategy to protect ferritin from proteolytic degradation. The authors showed that the ferritin cages were protected from degradation by protease when encapsulated in the microgels, and could be rapidly released under different environmental conditions, such as acidic pH. While not used for vaccination in this study, this platform has potential as a vaccine platform in the future.

Formulation of biologically derived nanoparticles into larger nanoparticle systems, rather than microparticles, has also been used to enhance immunogenicity (Rami et al., 2021; Teng et al., 2021). Gold nanocages (AuNCs) were recently used as a carrier for foot-and-mouth disease VLPs (Teng et al., 2021). AuNCs are porous, hollow structures capable of loading smaller nanoparticles into their interior. AuNC-VLP complexes were shown to promote a greater inflammatory response in mice than VLPs alone, agreeing with other studies that gold particles have an adjuvant effect *in vivo* (Niikura et al., 2013). Additionally, when used to immunize guinea pigs, the AuNC-VLPs showed a protective effect, generating neutralizing antibody titers with only one injection.

In pre-clinical animal models, formulation of biologically derived nanoparticles into larger, hybrid vaccine platforms has been shown to provide additional adjuvanticity and controlled release, generating a much greater immune response than that of the nanoparticles alone. In fact, these hybrid platforms can act as highly effective vaccines that may be administered orally or intranasally, rather than by injection. Needle-free vaccination is of great value, reducing the need for trained personnel and the occurrence of bloodborne pathogens such as hepatits B and HIV across the world. From these studies, it is evident that biologically derived hybrid platforms may be able to meet this need to develop effective single-dose, needle-free vaccines.

## Conclusion

To mount a protective immune response against an antigen, two main signals are needed: 1) repeated, high density motifs to strongly crosslink BCRs and 2) PAMPs for activation of B cells and T cells. Furthermore, nanoparticles within the size range of 20–200 nm will directly transport to the lymph (Manolova et al., 2008; Singh, 2021), and spherical particles smaller than 500 nm are preferentially taken up over larger particles of other shapes by APCs (Niikura et al., 2013). Biologically-derived nanoparticles naturally contain both PAMPs and repetitive surface structures capable of activating an effective immune response, and are most often smaller than 500 nm in size, making them promising vaccine platforms. These nanoparticles have many other useful properties, including biodegradability, self-assembly, and use of industrial processes already in place, allowing relatively simple scale-up from benchtop to large-scale manufacturing. Due to their native size, they are especially suited for efficient transport and localization to the lymph node, either via direct transport through the fenestrated lymphatic vessels, or through uptake by APCs. In comparison to whole-pathogen vaccines, such as live-attenuated and inactivated vaccines, biologically-derived nanoparticles have the potential for more favorable safety profiles, as they are non-infectious and unable to replicate; therefore, incomplete inactivation or pathogenic reversion is not a concern. Genetic modification or chemical linkage allows surface display of many heterologous antigens, and novel coupling strategies such as sortase-mediated conjugation (Thérien et al., 2017) and Addvax (Weyant et al., 2021) provide additional flexibility to further modify these highly versatile platforms for use against various diseases.

Here, we have reviewed three classes of biological nanoparticles (VLPs, OMVs, and protein cages) as vaccine platforms. As VLPs directly mimic native viruses, these nanoparticle systems can induce strong, T-independent stimulation of B cells through BCR crosslinking (Bachmann et al., 1993; Fries et al., 2021). VLPs also contain viral PAMPs, such as nucleic acids, that help activate, in many cases, and an adjuvant-free protective immune response (Venter et al., 2011; Li M. et al., 2021; Ortega-Rivera et al., 2021; Royal et al., 2021; Warner and Frietze, 2021). Additionally, if they are made in bacteria, VLPs may contain some bacterial components that further promote a protective response without use of classical alum adjuvants (Mohsen et al., 2021, 2022). As OMVs are directly derived from bacteria, they display many PAMPs that VLPs do not contain, such as LPS, flagellin, and lipopeptides, and making them self-adjuvanting. This property has enables OMVs to generate a protective immune response in various animal models for multiple diseases (Chen et al., 2010; Rosenthal et al., 2014; Kuipers et al., 2015; Watkins et al., 2017b; Klouwens et al., 2021). Protein nanocages, such as those derived from ferritin, also show promise as novel vaccine platforms. The repetitive surface properties of these compounds, their size, as well as their ease of modification, through either genetic or chemical means, makes them a very interesting platform capable of eliciting strong memory responses, and protection against viral challenge, even without the presence of adjuvant (Qi et al., 2018; Wei et al., 2020; Qu et al., 2021b).

The success of biologically-derived nanoparticles as vaccine platforms is evident in their clinical usage. Already, there are multiple VLP- and OMV-based vaccines on the market, and protein nanocage vaccines based on ferritin are currently in clinical trials. While controlled-release hybrid vaccine delivery platforms are merely in their infancy, these new techniques provide an exciting view of the potential for next generation vaccine technologies.
